# Quality of Life and Satisfaction from Career and Work–Life Integration of Greek Dentists before and during the COVID-19 Pandemic

**DOI:** 10.3390/ijerph19169865

**Published:** 2022-08-10

**Authors:** Maria Antoniadou

**Affiliations:** Dental School, National and Kapodistrian University of Athens, 11527 Athens, Greece; mantonia@dent.uoa.gr

**Keywords:** COVID-19, dental professionals, personal accomplishment, career satisfaction, psychological impact, quality of life, stress, burnout

## Abstract

Quality of life (QoL) of dental professionals is a basic parameter of the quality of dental services (QS), a fact well-documented before and during the COVID-19 pandemic in different countries. This study is a comprehensive, cross-sectional survey aimed to explore possible differences in satisfaction from career and work-life integration, as well as QoL in a sample of Greek dentists during the second lockdown in March 2021. Methods: 804 dentists from the vast metropolitan area of Athens and Piraeus selectively responded by completing a self-reported questionnaire based on: the Copenhagen Questionnaire (CQ) for assessing work stress; the Maslach Burnout Inventory-Human Service Survey (MBI-HSS) for evaluating personal accomplishment; and the Quality-of-Life work scale (ProQOL-CSF). Data were analyzed using the independent *t*-test, ANOVA, Pearson’s correlation, and multiple linear regression. Results: QoL and career satisfaction were significantly diminished during the pandemic. Career satisfaction despite the pandemic was overall influenced in tandem by age (b = 0.427, *p* = 0.001), marital status (b = 0.276, *p* = 0.021), and inversely by years of practice (b = −0.330, *p* = 0.007) and income (b = −0.221, *p* = 0.015). Satisfaction from the work–life integration was influenced before the pandemic by marital status (b = 0.255 *p* = 0.013), years of practice (b = −0.371, *p =* 0.0001), while gender, years of practice, age, higher education, and income played a significant role during the pandemic. QoL was impacted before pandemic by age (b = −1.007, *p* = 0.001), number of children (b = −1.704, *p* = 0.018), and higher degree (b = −1.143, *p* = 0.001), while during the pandemic by gender (b = −0.582, *p* = 0.002), number of children (b = 0.469, *p* = 0.037), higher degree (b = 0.279, *p* = 0.009), and years of practice (b = −0.523, *p* = 0.0001). Males were more prone to low QoL, and dissatisfaction with career and work–life integration, during the pandemic. Income is a predictor of career satisfaction despite the pandemic. Personal resources through deep human relationships, higher education, beliefs, and values can offer a resilience shield against professional difficulties in periods of unexpected stressful events.

## 1. Introduction

Quality of life (QoL) is closely related to professional life and is strongly suggested to be assessed in occupational health studies [1]. Dentistry could be a desirable career that, according to the public’s understanding, can bring a high-income reputation and good QoL [2]. Instead, high levels of job dissatisfaction and low QoL were reported in dentists, before [3,4,5] and during the COVID-19 pandemic [6]. Delivering oral health care is generally characterized by aspects likely to influence positively job satisfaction and QoL of dentists such as good patient relationships, respect and acceptance, delivery of care, high staff quality, professional relationships, and professional environment, or negatively such as low productivity, high-stress level, low salary and other economic issues, lack of resources, equipment shortage, extensive working hours, absence of free personal time, or low self-esteem [2,4,7]. Other factors negatively influencing the QoL of dental professionals are demographic characteristics such as the younger age, male gender, student status, high job-strain/working hours, those enrolled in clinical degree programs, and certain personality types [8].

It is a common truth that dentists have always been exposed to occupational stress with a high risk of burnout, which is strongly associated with job dissatisfaction, low QoL [7,9,10], and early retirement [7,11]. During the last few years, the COVID-19 pandemic has changed the dental routine by bringing a high risk of aerosol contamination into dental procedures and the urgency of developing innovative safety protocols in dental practices [12,13], which has caused extra stress, burnout, career dissatisfaction, and low QoL [7,14]. Moreover, through this period, dentists, especially in the private dental sector, were facing extended responsibilities and demanding economical reinforcement of the business as patients were reluctant or unable to spend [15,16,17,18]. During the pandemic, factors such as the female gender, the age being 50–59 years, being immune deficient or chronically ill, the pandemic being considered a financial hazard [16], or information overload [17] were the main causes of QoL worsening. In addition, the absence of personal time, the fear of COVID-19 contamination, and the workload in the dental office were important factors of personal judgment along with the risk of practicing unsafely dental procedures [19]. All the above lead to a lowering of the quality of dental services [5,15] and initiate a vicious circle of disappointment and distress for dental professionals affecting QoL [7].

Quality of life (QoL) is addressed by the World Health Organization (WHO) as “an individual’s perception of their position in life in the context of the culture and value systems in which they live and in relation to their goals, expectations, standards, and concerns” [20]. It is, therefore, a broad-ranging concept incorporating, in a complex way, the person’s physical health, psychological state, level of independence, social relationships, personal beliefs, and their relationships with salient features of the personal and professional environment. This definition suggests that QoL refers to a subjective evaluation, which cannot be equated simply with the terms “health status”, “lifestyle”, “life satisfaction”, “job satisfaction”, “mental state”, or “well-being”. Before the pandemic, different authors have tried to address and measure the qualities of QoL in terms of its impact on personal health, family life and activities, housekeeping, relationships with relatives, financial situation, professional life, and mental well-being [7,8,9,10,11]. Consequently, reduced professional fulfillment and job/career dissatisfaction were independently reported in many studies [21,22,23] based on personal health, social, cultural, and environmental factors that are presumed to influence subjective overload and psychological distress in different countries [24,25]. Finally, musculoskeletal disorders faced by dental professionals during the pandemic were also considered a main source of anxiety and low QoL, requiring an urgent holistic approach [26].

So, even though there is documentation on dentist’s QoL discrepancies almost all over the world, in this study, the relevant impact of the pandemic on a sample of Greek dentists was investigated. Different factors seem to influence QoL in dental professionals among countries during the pandemic and Greece was one of the least affected countries [27], up to the time of the study. Thus, in this paper, we aim to investigate factors affecting satisfaction from career and work–life integration as well as overall QoL in a sample of Greek dentists, during March–April 2021. More specifically, we searched the potential effect of different demographic variables, such as social and economic status, gender, age, level of education, and working experience, as well as ethical and practical limitations of dental services. A secondary goal is to provide awareness of the importance of QoL on the well-being of dentists and the emerged quality of dental services.

## 2. Materials and Methods

### 2.1. Questionnaire of the Study

QoL is a multi-dimensional concept based on a complicated causality of the mutual relationship of its indicators (variables). As it is true for all concepts, it is not actually measurable. What can be measured are the indicators or domains (groups of indicators) at the level of the individual as well as at the level of society or a specific professional field; it also relates to the assessment of “living good human lives” and evaluation of external environment. Like probably in all other relevant concepts, QoL also misses standardized unified methodology and terminology [28]. 

The questionnaire of the study was then based on the following questionnaires assessing different indicators: (1) Personal accomplishment (PA) was assessed using the 22-point questionnaire of Maslach (Μaslach Burnout Inventory-MBI-HSS) [29], which is extensively used in healthcare studies [30,31,32,33]; (2) “The Copenhagen Burnout Questionnaire (CBI)” [34] was also used to evaluate job satisfaction as discussed in studies of staff working in difficult medical settings [35], and (3) Quality of life was estimated for all participants by means of the Professional Quality of Life Scale version 5 (ProQOL-5) [36] also reported elsewhere [37].

The questionnaire was specifically developed for the study, translated by authenticated translators and pilot tested on 20 dentists to assess the comprehensibility and usability of all included questions [7]. The final survey version was uploaded to a Google Forms page and the link was sent with all relevant information of completion and consent forms to the secretariat of the two main dental associations in the vast area of metropolitan Athens, Greece, to forward to members.

Dentists who marked “strongly agree” or “agree” in response to the survey items, “Would you like any of your children to follow your profession? (Even if you don’t have children now)”, “If you were asked today, would you choose the dental profession?”, “I believe that I can make a difference for the improvement of society through the dental profession” were satisfied with their career. Those reported, “strongly agree” or “agree” in response to the survey items, “I was happy before the pandemic”, “I think I do not work long hours, and I have a personal life”, “ I am pleased because I feel able to evolve through my profession as a person and as a scientist”, “I am the person I have always wanted to be”, were respectively considered satisfied with the work–life integration in the statistical models followed in the study [7].

### 2.2. Sample

The questionnaire was approved by the scientific board of the dental associations of Athens and Piraeus, which gave permission via an email approval (protocol number 704, 17 April 2021). Following consent, the secretariat of both associations twice sent the invitation with the link of the Google Forms questionnaire and relevant information through an email message, from 20 March to 30th of April 2021. Inclusion criteria were: (a) active member of one of the two dental associations (meaning still working not retired and paying an annual subscription to the association), (b) practicing dentistry in Greece, and (c) being tax informed only in Greece. The exclusion criteria were: (a) dental hygienists, (b) auxiliary dental personnel, (c) dental students, (d) dentists working abroad, (e) dentists being taxed abroad, and (f) dentists not fulfilling association membership’s issues. The total number of members corresponding to criteria was N1 = 6300 dentists. This number corresponds to almost 46% of the population of dentists in Greece (N2 = 13.668) [38]. This high percentage augments the strength of the sample [39]. The privacy of participants was guaranteed through the online form of the questionnaire and every participant had the same chance to enroll in the survey by filling out the questionnaire. 

### 2.3. Statistical Analysis

The outcome variables were the percent frequencies and the mean scores for each survey question or summary scores of questions with similar content. The data were normally distributed according to Shapiro–Wilk test for normality (*p* > 0.05). Two types of inferential analysis were conducted; first, we assessed the potential differences in questions’ frequencies before and during the pandemic using Cochran’s Q test. In addition, multiple linear regression models were applied, having as predictors, the demographic characteristics of the sample and survey questions of interest, and as outcome variables, the survey questions mean scores. All reported probability values (*p* values) were compared with a significance level of 5% (*p* < 0.05). The analysis of coded data was carried out using the IBM SPSS Statistics for Windows, Version 27.0. Armonk, NY, USA: IBM Corp. 

## 3. Results

A total of 804 valid responses were gathered (response rate = 12.8% of the 6300 dentists in the sample). Despite the low number of filled questionnaires, the internal consistency of the survey was very satisfactory (Cronbach’s alpha: 0.81) [7,39]. Demographic analysis of the sample can be seen in Table 1.

More specifically, in Table 2, predictors of job satisfaction during the pandemic are presented. Answers to, “during the pandemic I feel trapped from my work in the dental office” were affected by gender. Males were more prone to answer positively to the question (b = 0.221, *p* = 0.023) while age (b = −0.257, *p* = 0.0001), number of children (b = −0.335, *p* = 0.004), and existence of another higher degree (b = −0.144, *p =* 0.009) affected it inversely. This means that the older the dentist, the more children they have, and the higher their educational status (existence of another higher degree), the less possible it is for them to feel trapped in their work in the dental office during COVID-19. Concerning answers for “during pandemic I like my job”, gender was again a predictor of a negative answer (for males) (b = −0.312, *p =* 0.0001), while years of practice (b = −0.162, *p =* 0.001), age (b = 0.144, *p =* 0.0001), and higher degree (b = 0.135, *p* = 0.005) were positive predictors. This means that the older and more educated the dentist, the more positively they would answer this question. Satisfaction from professional performance during the pandemic was inversely affected by years of practice (b = −0.121, *p* = 0.008). The more years in the profession the less satisfied a dentist was in our sample from his performance during the pandemic. 

Furthermore, answers to the question, “My remuneration is satisfactory for the work I do and the responsibilities I take on during the pandemic” were affected in tandem by age (b = 0.133, *p* = 0.001) and the existence of a higher degree (b = 0.215, *p* = 0.0001), while they were inversely affected by income (b = −0.009, *p* = 0.007). This means that the older the dentists and the more educated they are, the more satisfied they might be with their remuneration during the pandemic, while the less income they have annually, the less satisfied they may be with their remuneration during the pandemic. When asked if they would like any of their children to follow the profession (even if they do not have now), males were more prone to answer negatively (b = −0.219, *p* = 0.019), while married dentists answered positively (b = 0.114, *p* = 0.035). In addition, years of practice (b = −0.148, *p* = 0.009) and income (b = −0.114, *p* = 0.006) inversely affected the relevant answers with age in tandem (b = 0.165, *p* = 0.0001). “If you were asked today, you would choose the dental profession” was affected in tandem by age (b = 0.163, *p* = 0.0001) and inversely by years of practice (b = −0.121, *p* = *0*.034). “I’m happy during the pandemic” was less likely to have a positive answer in males (β = −0.196, *p* = 0.023). Number of children affected the question in tandem (b = 0.223, *p* = 0.031), while years of practice, inversely (b = −0.241, *p* = 0.0001).

In Table 3, predictors influencing job satisfaction before the pandemic are presented. “I was happy before the pandemic” was affected inversely by years in the profession (b = −0.189, *p* = 0.0001). Thus, the more years in the profession the less happy the dentist was before the pandemic. “Before the pandemic I was happy by my remuneration and the responsibilities I was taking on” was affected in tandem by the existence of a higher degree (b = −0.168, *p* = 0.0001). Answering positively, “I have beliefs/values/principles that support me to continue my work regardless the period of the pandemic we are experiencing” was affected by age (b = 0.060, *p* = 0.027). The older the dentist, the more likely they were to have found and organize their spiritual and ethical base that helps them overcome obstacles. When asked, “I am pleased because I feel able to evolve through my profession as a person and as a scientist regardless the period of the pandemic we are experiencing“, answers were affected in tandem by age (b = 0.126, *p =* 0.0001)and the existence of a higher degree (b = 0.114, *p* = 0.005), while being inversely affected by the years of practice (b = −0.109, *p* = 0.012) and income (b = −0.067, *p =* 0.034). “I am the person I have always wanted to be regardless the period of the pandemic we are experiencing” was influenced positively by age (b = 0.077, *p* = 0.026) and increased in married dentists (b = 0.095, *p* = 0.022). “I believe that I can make a difference for the improvement of society through the dental profession regardless of the period of the pandemic that we are experiencing” is affected positively by age (b = 0.098, *p* = 0.01).

Additionally, satisfaction from the career was influenced in tandem by age (b = 0.427, *p* = 0.001) and was increased in married dentists (b = 0.276, *p* = 0.021), while the years of practice (b = −0.330, *p* = 0.007) and income (b = −0.221, *p* = 0.015) inversely affected satisfaction. In addition, satisfaction with the work–life integration was increased in married professionals (b = 0.255, *p* = 0.013) and was inversely affected by years of practice (b = −0.371, *p* = 0.0001), as shown in Table 4.

In Table 5, the total score of professional quality of life (ProQOL) is presented. More specifically, QoL before the pandemic was influenced inversely by age (b = −1.007, *p =* 0.001), number of children (b = −1.704, *p* = 0.018), and the existence of a higher degree (b = −1.143, *p* = 0.001). This finding suggests that ProQOL is diminished while age, number of children, and educational level are augmented. During the pandemic, ProQOL was influenced by gender, thus decreasing in males (b = −0.582, *p* = 0.002) and was also inversely affected by years of practice (b = −0.523 *p* = 0.0001). However, the number of children and the existence of another higher degree (b = 0.279, *p* = 0.009) positively marked the phenomenon (b = 0.469, *p* = 0.037).

Overall, 725 dentists (90.2%) of the sample answered “agree” or strongly agree” with the phrase, “I have beliefs/values/principles that support me to continue my work despite the difficulties of the pandemic”; 622 dentists (77.4%) with the phrase“, I am pleased because I feel able to evolve through my profession as a person and as a scientist”; and 502 dentists (62.5%), with the phrase “I am the person I have always wanted to be”. In addition, 497 dentists (61.9%) reported as above on the phrase, “I believe that I can make a difference for the improvement of society through the dental profession”. Finally, 480 dentists (59.7%) would agree or strongly agree with the phrase, ”If you were asked today, would you choose the dental profession?”, and 316 dentists (39.3%) with the phrase, “Would you like any of your children to follow your profession? (even if you don’t have children now)” (data not shown, available upon request).

## 4. Discussion

During the pandemic, the continuing presence of safety issues and subsequent closures caused a constant fear of performing dentistry. It also posed a continued threat to the economic survival of dental businesses in Greece [7,13,15] and worldwide [40,41,42,43,44]. Economic problems were a contributing factor to low QoL before the pandemic [28], while they seemed to also play an important role during COVID-19 in our study and elsewhere [7,41,42,43]. Certain commentators, including Nobel laureate Amartya Sen, have questioned though the equivalence of economic status with the status of QoL [45], and so far, different predictors of QoL have been investigated [7,28].

As such, our findings reinforce the impact of higher education levels in assessing QoL for dentists during the COVID-19 pandemic. As already mentioned, education helps the increase in positive feelings, better perceptions of financial resources, effective estimation of physical environment, opportunities for acquiring information and skills [46], good health status, and probability of being married, all well-known predictors of high QoL [47,48,49,50]. In our study, high education level could not guarantee overall QoL before the pandemic, but it did so for all sexes during the pandemic through the increase in remuneration [51,52,53,54]. Income has generally been found to have a positive and statistically significant relationship with high QoL [55,56]. In our data, two out of three highly educated dentists were more prone to succeed financially during the pandemic, a fact that corresponds consequently to the rise of overqualified professionals in the country [57,58]. Negative beliefs or inability to receive the correct payment was also a significant source of anxiety and disappointment in the profession during the pandemic, in our study and elsewhere [41,42]. However, although dentists can struggle with economic matters, they may remain highly satisfied with their careers [2,59], as derived also from our data where two out of three dentists believe they can make a difference in the improvement of society through the dental profession and almost two out of three dentists would choose again the dental profession if they had the chance. Additionally, two out of three married dentists in our study certainly appreciated satisfaction from their career and work–life integration during the pandemic, possibly by experiencing stability in their roles, something reported to be generally important in QoL [60]. Married people in our study remain more satisfied with career and work–life integration than unmarried people, but the size of their advantage declines among men because unmarried men’s life satisfaction has generally increased lately [50]. Of course, during the pandemic and the subsequent closures, the courtesy of being free of relationships was diminished for both married and unmarried men. Gender itself seems to play an additional significant part in overall QoL, mainly during the pandemic than before. Two out of three men in our study were feeling more trapped in the dental office, possibly because economic problems had already dissatisfied them as mentioned before [61]. They had more to lose from joblessness (especially in terms of loss of self-esteem), something already mentioned in other studies even before the pandemic [62]. This broad pattern of comparatively large, non-pecuniary effects of gender, marriage, and loss of income on dentists’ QoL in our study holds also across different data sets and analytical methods [49,61]. Ιn our study too, one male dentist out of three expressed the wish for their children not to follow the dental profession. The same analogy exists despite gender in our sample, showing that gender is not a predictor for this specific matter. Furthermore, it is suggested elsewhere that job satisfaction may be significantly and positively predicted by weekly hours of work without significant differences according to gender [63] or appositively women, to announce lower QoL, having to address many different roles in and outside the house [61]. In our study, though, during the pandemic, expressed happiness decreased only in males, possibly since they were feeling trapped in and out of the dental office. Having children, also affected QoL in our study, more during the pandemic than before, possibly because children seem to absorb emotional and economic stress. Although the number of children can be an inverse predictor of physical burnout and exhaustion during the pandemic [7], it certainly affects positively the psychological health and gives a purpose in life, thus affecting QoL [64]. Before the pandemic most evidence on the relationship between having children and QoL suggested that parents were reporting lower QoL or reported the same level of QoL as non-parents [49,65,66]. One likely explanation for this was the negative impact of children on expenses and financial satisfaction affecting QoL, which was a common finding across many different countries around the world [67,68]. So, although children put demands on day-to-day parents’ emotions, people still regard them as a positive contribution when providing a cognitive evaluation of QoL [68], as was the case in our study. Further, age is one of the most widely researched predictors of job satisfaction [69] and was also addressed as an important inverse factor for total QoL in our study before the pandemic. Age is negatively related mainly to job satisfaction, both directly and indirectly, via job burnout [7,70]. During the pandemic, though, it affected positively our participants’ faith in the dental profession and the remuneration they could receive since one out of three dentists over 51 years of age feel more certain to acquire bigger remuneration. They also seem to be more certain of their dental role and more satisfied with their career, a view also reported previously [2]. It seems that wisdom and experience acquired through the years support and strengthen the belief and value system that can make dentists correspond to stressful situations [7]. Almost all dentists (three out of three) over 51 years of age in our sample use this system to support themselves in difficulties.

It is obvious from our data that over the course of a dental career the public attitude to dentistry or safety issues during unexpected events such as the pandemic can wear professionals down. Dentists could counteract this by remembering why they chose dentistry or why they stayed in the profession in the first place and drive toward the icon of the person they have always wanted to be. They could also become inspired by thinking about dentists they have admired, and by focusing on the real benefits of being in this profession—from the daily achievements at work to the opportunities that the rewards of dentistry can bring to their life outside work [71] through valued human relationships. As is well-known, self-care and quality personal and professional time have the potential not only to minimize harm from stress, burnout, physical, and psychological distress, but also to promote personal and professional QoL [23,41]. Thus, state and professional organizations should work on positive psychology projects by enhancing a positive attitude to life and work for their members. They should also provide activities, assessments, and resources that address specifically self-care issues and physical status relevant to work in the dental field [26,72,73].

The cross-sectional design of the study does not allow for further direct identification of causal factors of satisfaction from career and work–life integration or QoL. Moreover, the pandemic is not over, so there is the possibility that dentists’ responses before the pandemic can be biased. Thus, further investigation of the matter in current pandemic conditions is in our future research plans. Although we report a low response rate, this does not affect the internal consistency of the survey if we also consider the Cronbach’s alpha index retrieved, and the fact that 46% of the total population of dentists in Greece was the study sample. It seems that one out of ten dentists in this sample responded to our survey, which is a very satisfactory impact compared to relevant studies. Further, at the time the questionnaire was sent, dentists were preparing to reopen after a year’s closure, and obviously, they were quite busy, reluctant, or unable to spend time, which is also an issue for a future investigation. Finally, more research is needed on how beliefs, values, spiritual principles, or practical tactics can reinforce younger and middle-aged dental professionals’ attitudes on the importance of the dental profession in their personal and social life.

## 5. Conclusions

Quality of life is an important professional issue that needs further research both nationally and worldwide since it correlates to dentists’ perception of career satisfaction, social effectiveness, productivity, and remuneration. Education is the main predictor for QoL, followed by remuneration, social status, gender, and age. Greek male dentists can be more prone to dissatisfaction from their career and work–life integration, or a sense of low QoL during the pandemic. Remuneration is a predictor of career satisfaction despite the pandemic. Personal resources through deep human relationships, higher education, beliefs, and values can offer a resilience shield against professional difficulties in periods of unexpected stressful events.

## Figures and Tables

**Table 1:** Demographic characteristics of the respondents (n = 804).

Title 1	Title 2	Title 3	Title 4
Sex	Categories	Data	Percentage
	Women	442	44.92%
	Men	361	55%
	Other	1	0.1%
Age	Up to 30 years	74	9.2%
	31–40	138	17.2%
	41–50	263	32.7%
	51–60	233	29%
	60 and above	96	11.9%
Personal relationship status	Married	521	64.8%
	Unmarried	189	23.5%
	Divorced	70	8.7%
	Other	24	3%
Having children	No children	247	30.7%
	1–2	485	60.3%
	3 and more	72	9%
Dental educational status	Basic dental education	506	62.9%
	Master’s degree	228	28.4%
	PhD degree	70	8.7%
General educational status	Only dentistry diploma	666	82.8%
	Diploma in another science	138	17.2%
Dental clinical practice	General dentistry	610	75.9%
	Esthetic dentistry	26	3.2%
	Oral surgery	9	1.1%
	Endodontics	33	4.1%
	Orthodontics	31	3.9%
	Pedodontics	34	4.2%
	Periodontology	21	2.6%
	Prosthetics	29	3.6%
	Stomatology	4	0.5%
	Other	7	0.9%
Years in the profession	1–10 years	162	20.1%
	11–20 years	244	30.3%
	21–30 years	219	27.2%
	31 and above years	179	22.3%
Professional field	Private office	633	78.7%
	Public hospital	31	3.9%
	University	14	1.7%
	Private office + freelancer	28	3.5%
	Only freelancer	27	3.4%
	Working with another dentist	34	4.2%
	Employee	30	3.7%
	Other form	7	0.9%
Staff status	No employee	453	56.3%
	1 employee	170	21.1%
	2–3 and more	65	8.1%
	4 and above	24	3%

**Table 2:** Job satisfaction predictors’ analysis during the pandemic (multiple linear regression modeling).

	Job Satisfaction Outcome Variables				
		B	95% CI for B	P	Association
Predictors	*“During the pandemic I feel trapped from my work in the dental office* *”*				
	Gender	0.221	0.030 0.411	0.023	Increased in males
	Age	−0.257	−0.348 −0.165	0.0001	Negative (inverse)
	Number of children	−0.335	−0.562 −0.107	0.004	Negative (inverse)
	Higher degree	−0.144	−0.252 −0.036	0.009	Negative (inverse)
	*“During the pandemic I like my job”*				
	Gender	−0.312	−0.479 −0.146	0.0001	Decreased in males
	Age	0.144	0.064 0.225	0.0001	Positive (in tandem)
	Higher degree	0.135	0.041 0.230	0.005	Positive (in tandem)
	Years of practice	−0.162	−0.261 −0.062	0.001	Negative (inverse)
	*“During the pandemic I am satisfied with my professional performance”*				
	Years of practice	−0.121	−0.210 0.032	0.008	Negative (inverse)
	*“During the pandemic, my remuneration is satisfactory for the work I do and the responsibilities I take on”*				
	Age	0.133	0.053 0.212	0.001	Positive (in tandem)
	Higher degree	0.215	0.122 0.309	0.0001	Positive (in tandem)
	Income	−0.099	−0.172 −0.027	0.007	Negative (inverse)
	“*If you were asked today, you would choose the dental profession”*				
	Age	0.163	0.073 0.254	0.0001	Positive (in tandem)
	Years of practice	−0.121	−0.232 −0.009	0.034	Negative (inverse)
	*“I am happy during the pandemic”*				
	Gender	−0.196	−0.365 −0.027	0.023	Decreased in males
	Age	0.223	0.020 0.425	0.031	Positive (in tandem)
	Marital status	−0.241	−0.342 −0.140	0.0001	Negative (inverse)

**Table 3:** Predictors of career satisfaction issues before the pandemic (multiple linear regression modeling).

	Predictors Influencing Job Satisfaction before Pandemic				
Predictors	*“I was happy before the pandemic”*				
	Years in profession	−0.189	−281 −0.098	0.0001	Negative (inverse)
	*“Before the pandemic, my remuneration was satisfactory for the work I was doing and the responsibilities I was taking on”*				
	Higher degree	0.168	0.083 0.253	0.0001	Positive (in tandem)
	“*I have beliefs/values/principles that support me to continue my work despite the difficulties of the pandemic regardless the period of the pandemic we are experiencing”*				
	Age	0.060	0.007 0.114	0.027	Positive (in tandem)
	*“ I am pleased because I feel able to evolve through my profession as a person and as a scientist regardless the period of the pandemic we are experiencing”*				
	Age	0.126	−0.052 0.126	0.0001	Positive (in tandem)
	Higher degree	0.114	0.034 0.194	0.005	Positive (in tandem)
	Years of practice	−0.109	0.193 −0.024	0.012	Negative (inverse)
	Income	−0.067	−0.129 −0.005	0.034	Negative (inverse)
	*“I am the person I have always wanted to be regardless the period of the pandemic we are experiencing”*				
	Age	0.077	0.009 0.144	0.026	Positive (in tandem)
	Marital status	0.095	0.014 0.176	0.022	Increased in married
	*“I believe that I can make a difference for the improvement of society through the dental profession regardless of the period of the pandemic that we are experiencing”*				
	Age	0.098	0.024 0.172	0.01	Positive (in tandem)

**Table 4:** Predictors of professional satisfaction (multiple linear regression modeling).

	Satisfaction Issues				
		B	95% CI for B	P	Association
Predictor	**Satisfaction from the career** Summary score of *“Would you like any of your children to follow your profession? (even if you don’t have children now)” and ”If you were asked today, would you choose the dental profession?” and “I believe that I can make a difference for the improvement of society through the dental profession”*				
	Age	0.427	0.233 0.622	0.001	Positive (in tandem)
	Marital status	0.276	0.042 0.509	0.021	Increased in married
	Years of practice	−0.330	−0.571 −0.090	0.007	Negative (inverse)
	Income	−0.221	−0.398 −0.044	0.015	Negative (inverse)
	**Satisfaction from the work–life integration** Summary score of *“I was happy before the pandemic” and ”I think I do not work long hours and I have a personal life” and “I am pleased because I feel able to evolve through my profession as a person and as a scientist” and ”I am the person I have always wanted to be”.*				
	Marital status	0.255	0.054 0.457	0.013	Increased in married
	Years of practice	−0.371	−0.578 −0.163	0.0001	Negative (inverse)

**Table 5:** Predictors on total score of ProQOL (multiple linear regression modeling).

	Total Score of ProQOL				
		B	95% CI for B	P	Association
Predictor	*ProQOL score before the pandemic*				
	Age	−1.007	−1.574 −0.440	0.001	Negative (inverse)
	Number of children	−1.704	−3.112 −0.297	0.018	Negative (inverse)
	Higher degree	−1.143	−1.809 −0.477	0.001	Negative (inverse)
	*ProQOL score during the pandemic*				
	Gender	−0.582	−0.950 −0.214	0.002	Decreased in males
	Number of children	0.469	0.029 0.910	0.037	Positive (in tandem)
	Higher degree	0.279	0.071 0.488	0.009	Positive (in tandem)
	Years of practice	−0.523	−0.743 −0.304	0.0001	Negative (inverse)

## Data Availability

The data presented in this study are available on request from the corresponding author. The data are not publicly available due to ethical restrictions.
